# Case Report: First Report of Fatal *Legionella pneumophila* and *Klebsiella pneumoniae* Coinfection in a Kidney Transplant Recipient

**DOI:** 10.3389/fmed.2022.912649

**Published:** 2022-06-13

**Authors:** Maria Scaturro, Luna Girolamini, Maria Rosaria Pascale, Marta Mazzotta, Federica Marino, Giulia Errico, Monica Monaco, Antonietta Girolamo, Maria Cristina Rota, Maria Luisa Ricci, Sandra Cristino

**Affiliations:** ^1^Department of Infectious Diseases, Istituto Superiore di Sanità, Rome, Italy; ^2^European Society of Clinical Microbiology and Infectious Diseases (ESCMID) Study Group for Legionella Infections (ESGLI), Basel, Switzerland; ^3^Department of Biological, Geological, and Environmental Sciences, University of Bologna, Bologna, Italy

**Keywords:** Legionnaires' disease, *Legionella pneumophila*, *Klebsiella pneumoniae*, coinfection, incubation period

## Abstract

A very rare case of pulmonary *Klebsiella pneumoniae-Legionella pneumophila* coinfection in a double kidney transplanted man affected by the chronic renal disease is described. Cases of Legionnaires' disease with an incubation period of 14 days have rarely been documented. Despite the long period of hospitalization, typing of clinical and environmental *L. pneumophila* strains demonstrated that the patient's home water distribution system was the source of infection, highlighting that *Legionella* house contamination can be a hidden risk, especially for immune-compromised people.

## Introduction

Legionnaires' disease (LD) is an important public health threat caused by Gram-negative bacteria *Legionella pneumophila* (*Lp*), the species most frequently reported in infection cases (1). Most LD cases are community- or travel-acquired; however, nosocomial cases represent a great public health concern because of the high fatality rate (1). Most cases are susceptible individuals due to age, underlying diseases, abuse of alcohol, smoking, or immunosuppression (2). Water from different artificial aquatic environments, such as water systems in buildings and cooling towers, is the main reservoir of *Legionella* infection transmission by inhalation of infectious aerosols produced by showers and spa pools, as well as other devices producing aerosols. Although less common, micro-aspiration of contaminated water or direct contact with surgical wounds has been reported (3).

Here, we report an *Lp* and *Klebsiella pneumoniae* (*Kp*) coinfection case in an adult man affected by chronic renal disease.

## Case Description

**Figure 1:**
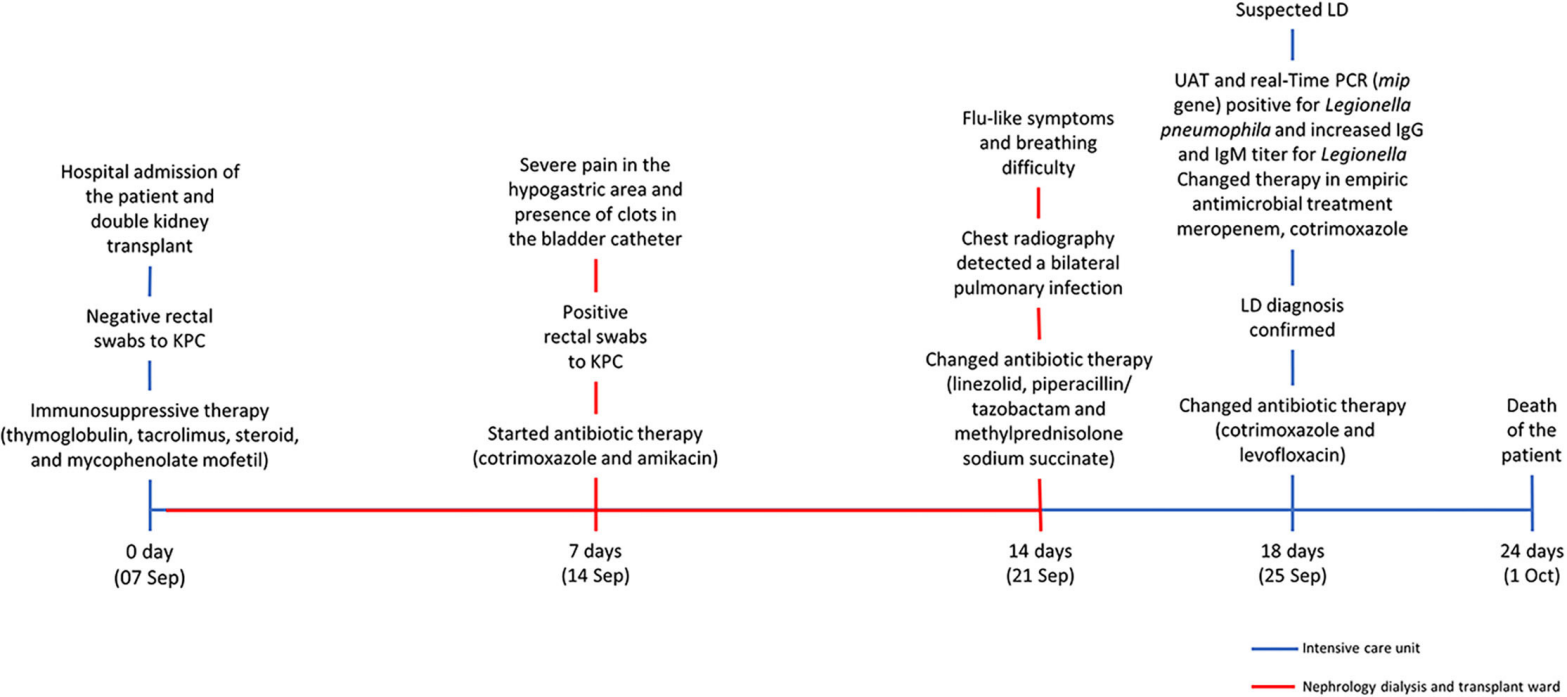
The hospitalization timeline and the clinical treatments.

A 59 years-old man with a background history of end-stage chronic renal failure was admitted to the hospital and on the same day underwent a double kidney transplant (7 September). Soon after surgery, he was moved to the intensive post-transplant care unit (ICU). The day after (8 September), he was transferred to the nephrology dialysis and transplant ward located in another building of the same hospital. Fourteen days after the surgery (21 September), he was transferred to ICU where he remained until his death (1 October). The timeline of hospitalization and clinical treatments are shown in Figure 1. Seven days after the surgery (14 September), because of severe pain in the hypogastric area, increased inflammation indices, and the presence of clots in the bladder catheter, rectal swabs were required. Because of the positive results for KPC-producing *Kp*, the patient was isolated, and an appropriate antibiotic therapy based on co-trimoxazole (strain with MIC = 40 mg/L) and amikacin (MIC = 8 mg/L, was undertaken. Noteworthy was that the analysis of rectal swabs, performed at the time of admission, was negative. Fourteen days after the surgery (21 September), chest radiography was performed because of flu-like symptoms and breathing difficulty and detected a bilateral pulmonary infection, and chest physical examination showed reduced vesicular murmur, SatO_2_ of 93%, and C-reactive protein of (CRP) 26. The patient was moved back to the ICU, and an antibiotic therapy based on a combination of linezolid with piperacillin/tazobactam (600 mg twice daily) was started, in association with previous treatment based on thyroglobulin (60 mg/die) plus glucocorticoids (methylprednisolone sodium succinate 40 mg twice daily).

Legionnaires' disease (LD) was suspected, and urinary antigen test (UAT) and IgG and IgM titers for *Legionella* were required. At the same time, a bronchoalveolar lavage (BAL) sample was cultured for aerobic bacteria and a real-time PCR assay for *Legionella* (*mip* gene target) was performed. The UAT and real-time PCR were positive, and the increased antibody titer was also observed (IgG = 354 U/ml; IgM= 1,153 U/ml), confirming the LD diagnosis; the health authority was immediately notified (25 September). The antibiotic therapy was then modified with meropenem (1gr/die), cotrimoxazole (3fl/ die), and levofloxacin (500 mg/die). Nevertheless, the patient got worse, and 6 days after the LD diagnosis (1, October) he died.

Ten lung tissue fragments, one blood, and one bile sample were collected post-mortem for *Legionella* culture, which was performed according to the Italian and Regional Guidelines for prevention and control of legionellosis (4, 5). Direct immune-fluorescence (MonoFluo™ Kit *Legionella pneumophila*; BioRad), detecting all *Lp* serogroups, and real-time PCR assay (6), specific for the *Legionella pneumophila* serogroup 1 (*Lp*1), were also performed. The real-Time PCR assay detected *Lp*1 in all lung tissue samples, while the direct immune-fluorescence test showed *Lp* in seven out of 12 samples tested, including the blood and bile samples. The culture of ten lung tissue samples revealed the presence of *Lp* colonies in two out of the 10 fragments analyzed, while *Kp* colonies were found in almost all the fragments.

*Kp* colonies were tested with the broth microdilution method to determine their *in vitro* sensitivity to a panel of 16 antibiotics. According to the EUCAST 2021 breakpoints these colonies resulted resistant to ertapenem, meropenem, and tigecycline and also positive to the *blaKPC* gene by PCR (7, 8). *Lp* colonies were also tested for antimicrobial susceptibility by broth microdilution method test according to EUCAST 2021 (9). The patterns of antibiotic susceptibility testing for *Lp*1 and *Kp* from lung fragments and rectal swab samples are shown in Table 1. To identify the source of *Lp* infection, the environmental investigation was promptly started at the patient's home and in the hospital wards where the patient was hospitalized. Overall 75 samples were collected in accordance with ISO 19458:2006 (10) as follows: 43 from hospital wards and 32 from the patient's home, including showers, jacuzzi, and kitchen sink faucet (Table 2). The samples were cultured in accordance with UNI EN ISO 11731:2017 (11), and the colonies were identified by the *Legionella* latex agglutination test (*Legionella* latex test kit; Thermo Fisher Diagnostic, Basingstoke, United Kingdom, and Biolife, Milan, Italy).

**Table 1:** *Legionella pneumophila* serogroup 1 (*Lp*1) and *Klebsiella pneumoniae* (*Kp*) antibiotic susceptibility from (a) lung tissue fragments and (b) rectal swab sample.

	***Legionella pneumophila*** **(*****Lp*****1)** **(a)**		***Klebsiella pneumoniae*** **(*****Kp*****)** **(a)**		***Klebsiella pneumoniae*** **(*****Kp*****)** **(b)**	
**Antibiotics pattern**	**MIC**	**Interpretation categories**	**MIC**	**Interpretation categories**	**MIC**	**Interpretation categories**
	**(mg/L)**	**(S/R)**	**(mg/L)**	**(S/I/R)**	**(mg/L)**	**(S/I/R)**
Azithromycin	0.06	S				
Amikacin			≤ 4	S	8	S
Amoxicillin / Clavulanic acid			≤ 64/2	R	≥32	R
Cefepime			>16	R	≥32	R
Ceftazidime			>64	R	≥64	R
Ceftazidime/avibactam			2/4	S		
Ceftriaxone			>4	R		
Ciprofloxacin	0.03	S	>1	R	≥4	R
Clarithromycin	0.03	S				
Colistin			1	S		
Doxycycline	4	S				
Ertapenem			>2	R	≥8	R
Erythromycin	2	S				
Gentamicin			≤ 1	S	≥16	R
Levofloxacin	0.03	S	>8	R		
Meropenem			8	I	≥16	R
Moxifloxacin	0.03	S				
Phosphomycin			8	S		
Piperacillin / Tazobactam			>128/4	R	≥128	R
Rifampicin	0.00046	S				
Tigecycline	32	S	1	R	4	R
Trimethoprim / Sulfamethoxazole			>8/152	R	40	S

*S, sensible; I, intermediate; R, resistant; according to EUCAST criteria (8, 9)*.

**Table 2:** Environmental sampling sites of water distribution systems, and *Legionella* concentration, identification, and typing.

	**Water output**	**Hot recirculating water**	**Hot water distal outlet**	**Cold water distal outlet**	**Swab samples**	**Total of positive sample**	***Legionella* range concentration (CFU/L)**	***Legionella* identification**	***Lp*** **1 typing**	
									**Monoclonal antibody (MAb)**	**Sequence Type (ST)**
Transplant ICU (*n* = 18)	2	2	10	2	2	4	300–30,000	*Lp1*and *Lp3*	*Lp*1, subgroup Oxford	1
Nephrology (*n* = 25)	2	2	11	6	4	5	100–5,000	*Lp1*and *Lp3*	*Lp*1, subgroup Oxford	1
Patient's home (*n* = 32)	2	1	10	13	6	10	100–2,800	*Lp1* and *L.anisa*	*Lp*1, subgroup Benidorm	664

Table 2 summarizes the *Legionella* water sample results. *Lp*1 was isolated both in the hospital and at the patient's home. *Lp*1 and *Lp*3 were discovered in the sink faucet and bidet of the patient's room toilet at concentrations ranging from 100 to 30,000 CFU/L in ICU and Nephrology wards, respectively. *Lp*1 and *L. anisa* contamination was identified throughout the patient's home, particularly in the kitchen sink faucet and in the two toilettes (10/32 positive samples).

Clinical and environmental *Lp*1 colonies were typed with monoclonal antibody (MAb) (12) and by sequence-based typing (SBT) (13). The *Lp* clinical strains, as well as the environmental strains isolated from the patient's home, specifically in the kitchen sink faucet, the shower, and the jacuzzi of one of the two toilettes, belonged to the subgroup Benidorm, Sequence Type (ST) 664, while the *Lp*1 colonies isolated from the hospital belonged to the subgroup Oxford ST1.

## Discussion

The case reported here is that of a very rare coinfection with *Lp* and *Kp* in a double kidney transplanted man affected by chronic renal disease. Indeed, concurrent or sequential coinfections of *Lp* with other pathogens have also been infrequently reported, mainly regarding viral coinfections (14, 15). A unique case of *Lp*-*Kp* coinfection had previously been reported in a young man who underwent a kidney transplant 4 years earlier and was healed with appropriate antibiotic therapy (16). However, according to the EU (17) and CDC (18) LD case definition, the case described by Dow and Chow was a probable case because it was diagnosed only by direct fluorescence with *Lp*-specific monoclonal antibodies, while the *Kp* infection was ascertained by culture.

*Kp* is an opportunistic pathogen that causes a broad spectrum of diseases and shows an increasingly frequent acquisition of antibiotic resistance. Multidrug-resistant infections have been documented worldwide as being caused by emerging major pathogens of international concern. By the production of extended-spectrum β-lactamases (ESBLs) and carbapenemases, hypervirulent *Kp* strains cause a variety of infectious diseases, including urinary tract infections, bacteremia, pneumonia, and liver abscesses (19). *Kp* colonizes various mucosal surfaces from the nasopharynx to the gastrointestinal mucosa, and in hospitalized patients, colonization rates in the gastrointestinal tract are higher (20). Additionally, *Kp*-colonized ICU patients have a higher risk of infection than non-carriers. The transition from colonization to infection is primarily due to impairment of host defense contributed by underlying diseases or immunodeficiency conditions. One of the factors that help to recognize a transition colonization-infection is positive culture. Although the patient's rectal swabs were negative for *Kp* at hospital admission, post-mortem examination of the lung tissue fragments revealed a massive presence of *Kp*, suggestive of lung colonization in the patient with severely immune-compromised conditions due to immunosuppressive therapy (thyroglobulin, etc.). Therefore, it is difficult to establish which of the two pathogens was the real cause of his death.

*Lp* could almost certainly have been the primary infection because the patient got infected at his own home. Inhalation of *Lp*-contaminated aerosols is usually followed by an incubation period ranging from 2 to 10 days before the onset of symptoms. In this case, the onset of symptoms 14 days after the admission strongly suggested a nosocomial LD case; however, only the MAb and SBT typing demonstrated that the patient acquired the *Lp*1 infection at home. These findings highlight the importance of performing *Legionella* culture on patients' respiratory secretions or postmortem lung tissues.

Although rarely observed, longer incubation periods are actually possible as already documented, mainly in immune-compromised patients (21). For this reason, according to the CDC case definition, also at the European level, the incubation period should be extended to 14 days (18).

Noteworthy, a very strange matter was that most probably the patient contracted the *Lp* infection simply by cleaning the jet breaker of the kitchen sink faucet as he used to do. Indeed, it has also been referred that the patient never used his jacuzzi or the toilette where *Lp*1 ST664 was found. In addition, home-acquired LD infections are increasingly documented (22, 23), and most likely, they could represent a high percentage of not investigated community-acquired LD cases, which remain without an identified source of infection, contributing to the underestimated rate of LD notification at Italian ad European levels.

## Conclusion

The case reported here highlights once more the risk of LD infection at home and the importance of considering an incubation period longer than 10 days to suspect LD and consequently to ascertain and assign the true source of the infection. Besides, since the susceptibility to LD of transplanted patients is well-documented, urinary antigens should be administered immediately upon the first appearance of symptoms to promptly treat patients. If LD is confirmed, appropriate and immediate antibiotic treatments able to counteract this severe and often fatal infection must be given. In addition, physicians caring for these patients, as well as all immunocompromised or elderly patients in general, should inform these more susceptible people about the risks of contracting LD from all aerosol-producing household appliances and provide them with basic information on how to prevent it. Finally, the reported case highlights the importance of isolating and characterizing *Legionella* from clinical samples, as well as focusing on the role of other sources of infection (e.g., home exposure) that, if not taken into account during the appropriate period of incubation, could interfere with case definition (nosocomial, travel, or community-acquired) and notification rate.

## Data Availability Statement

The raw data supporting the conclusions of this article will be made available by the authors, without undue reservation.

## Ethics Statement

Ethical review and approval was not required for the study on human participants in accordance with the local legislation and institutional requirements. The patients/participants provided their written informed consent to participate in this study.

## Author Contributions

MS, SC, and MLR conceived and designed the study and wrote the article. LG, FM, MRP, and MMa performed the experiments on environmental samples. GE, MMo, and AG performed the experiments on human samples. MCR revised the original draft of the manuscript and provided the Italian epidemiological data. All the authors read and agreed to the published version of the manuscript.

## Conflict of Interest

The authors declare that the research was conducted in the absence of any commercial or financial relationships that could be construed as a potential conflict of interest.

## Publisher's Note

All claims expressed in this article are solely those of the authors and do not necessarily represent those of their affiliated organizations, or those of the publisher, the editors and the reviewers. Any product that may be evaluated in this article, or claim that may be made by its manufacturer, is not guaranteed or endorsed by the publisher.
